# Successful treatment with nivolumab in a patient with lung adenocarcinoma complicated by pulmonary aspergilloma

**DOI:** 10.1111/1759-7714.13662

**Published:** 2020-09-17

**Authors:** Naoki Furuya, Koji Kojima, Hideki Marushima, Kazutaka Kakinuma, Akihito Tsunoda, Eriko Koda, Hajime Tsuruoka, Kohei Nishida, Takeo Inoue, Hisashi Saji, Masamichi Mineshita

**Affiliations:** ^1^ Division of Respiratory Medicine, Department of Internal Medicine St. Marianna University School of Medicine Kawasaki Japan; ^2^ Department of Chest Surgery St. Marianna University School of Medicine Kawasaki Japan

**Keywords:** Anti‐PD‐1 antibody, chronic infection, nivolumab, non‐small cell lung cancer, pulmonary aspergilloma

## Abstract

Immune checkpoint inhibitors (ICIs) are the key drugs used in patients with non‐small cell lung cancer (NSCLC). However, anti‐PD‐1 therapy might worsen chronic infection by reactivating the immune response to infectious diseases. Here, we describe a case of successful treatment with nivolumab in a patient with NSCLC complicated by pulmonary aspergilloma, which was safely treated by surgical resection before administration of nivolumab. In conclusion, to safely treat patients with locally limited chronic pulmonary aspergillosis (CPA), surgical resection should be considered before ICI therapy.

## Graphical abstract

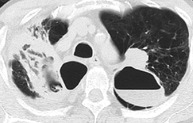

Anti‐PD‐1 therapy might worsen chronic infection by reactivating the immune response to infectious diseases. Here, we describe the first successful case of a patient safely treated with nivolumab for advanced stage NSCLC complicated by pulmonary aspergilloma.

## Introduction

Currently, immune checkpoint inhibitors (ICIs) are the key drugs used in patients with non‐small cell lung cancer (NSCLC). Nivolumab is one of the ICIs that is characterized by fully human IgG4 anti‐programmed cell death‐1 (PD‐1) monoclonal antibody.^1^ Nivolumab showed superiority for overall survival (OS) in a study comparing docetaxel in patients with advanced nonsquamous non‐small cell lung cancer (Non‐sq NSCLC).^2^ However, it has been reported that anti‐PD‐1 therapy might worsen chronic infection by reactivating the immune response to infectious diseases, especially in *Mycobacterium tuberculosis*,^3^ *Pseudomonas aeruginosa*,^4^ and *Aspergillus fumigatus*.^5^, ^6^ In general, medical oncologists might hesitate to administer anti‐PD‐L1 therapies in these patients. We herein report a successful case of treatment with nivolumab in a patient with NSCLC complicated by pulmonary aspergilloma.

## Case report

**Figure 1:**
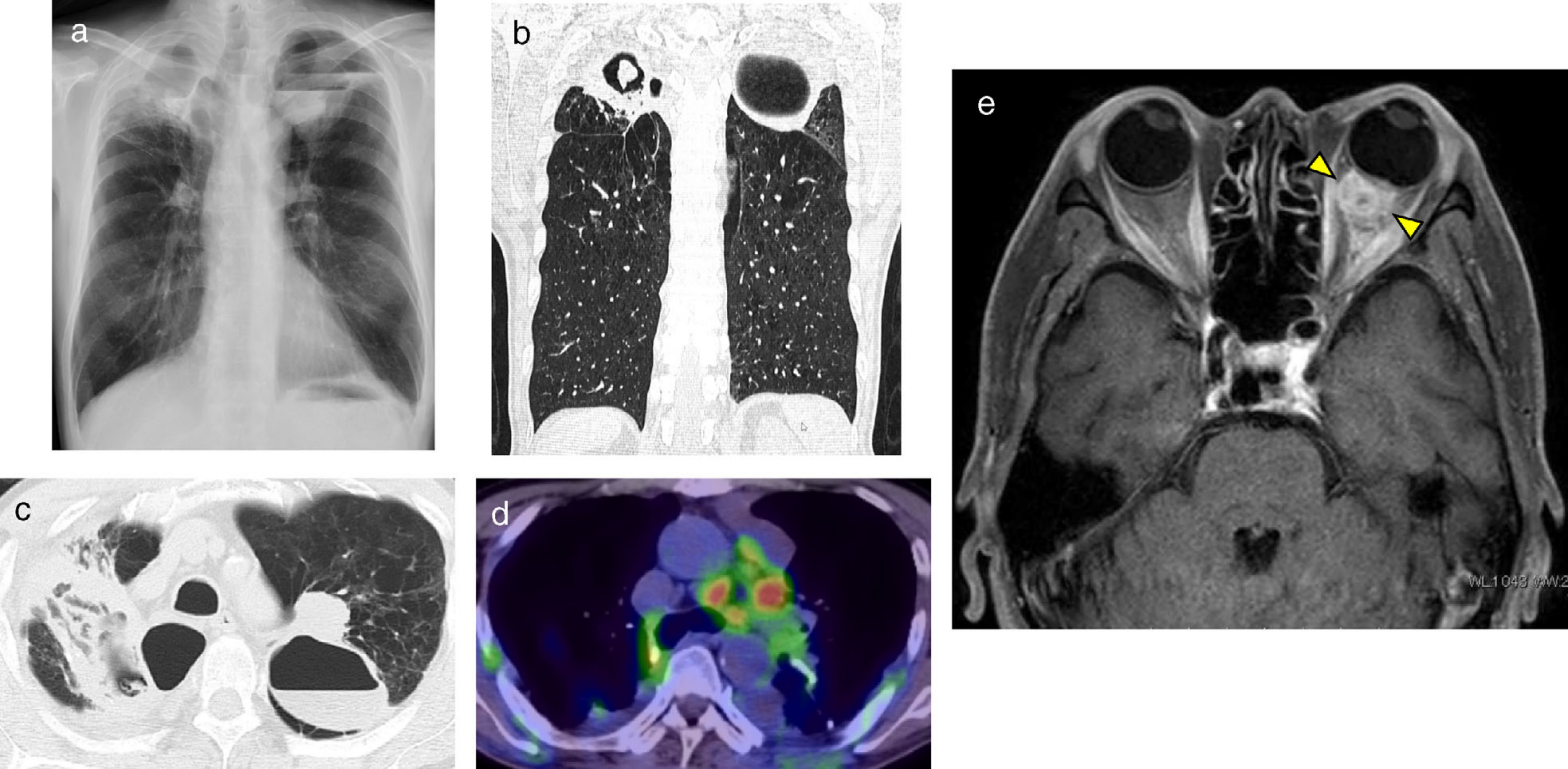
(**a**) Chest X‐ray from previous hospital. (**b**, **c**) Chest computed tomography (CT) revealed cavity formations in both upper lobes. (**d**) Positron emission tomography‐computed tomography (PET‐CT) scan showed high level uptake of FDG in the mediastinal lymph nodes. (**e**) Magnetic resonance imaging (MRI) of the head revealed an 11 mm nodule in the left choroid (arrows).

A 59‐year‐old male with a history of hemoptysis for two months was previously admitted to a different hospital where chest X‐ray had revealed an abnormal shadow in both upper lungs (Fig 1a). The patient had complained of a recent left visual field defect and was subsequently referred to our institution for a definitive diagnosis and treatment.

He was a heavy smoker (40 pack‐year). Eastern Cooperative Oncology Group (ECOG) performance status (PS) was 1. Computed tomography (CT) scan showed cavity formation in both upper lobes (Fig 1b,c). In the cavity of the right upper lobe, there was an 18 mm nodule which was suspected to be a fungus ball. There was a 30 mm tumor shadow adjacent to the left upper lobe cavity. Positron emission tomography‐computed tomography (PET‐CT) scan revealed high level uptake of fluorodeoxyglucose (FDG) in the upper lobe tumor shadow and mediastinal lymph nodes (Fig 1d). Magnetic resonance imaging (MRI) of the head showed an 11 mm nodule in the left choroid (Fig 1e). Tumor markers were elevated (CEA: 62.1 ng/mL) and β‐D glucan and aspergillus antigen were both positive. However, a relatively high amount of hemoptysis continued for one month, which made it difficult to perform bronchoscopy for pathological diagnosis.

**Figure 2:**
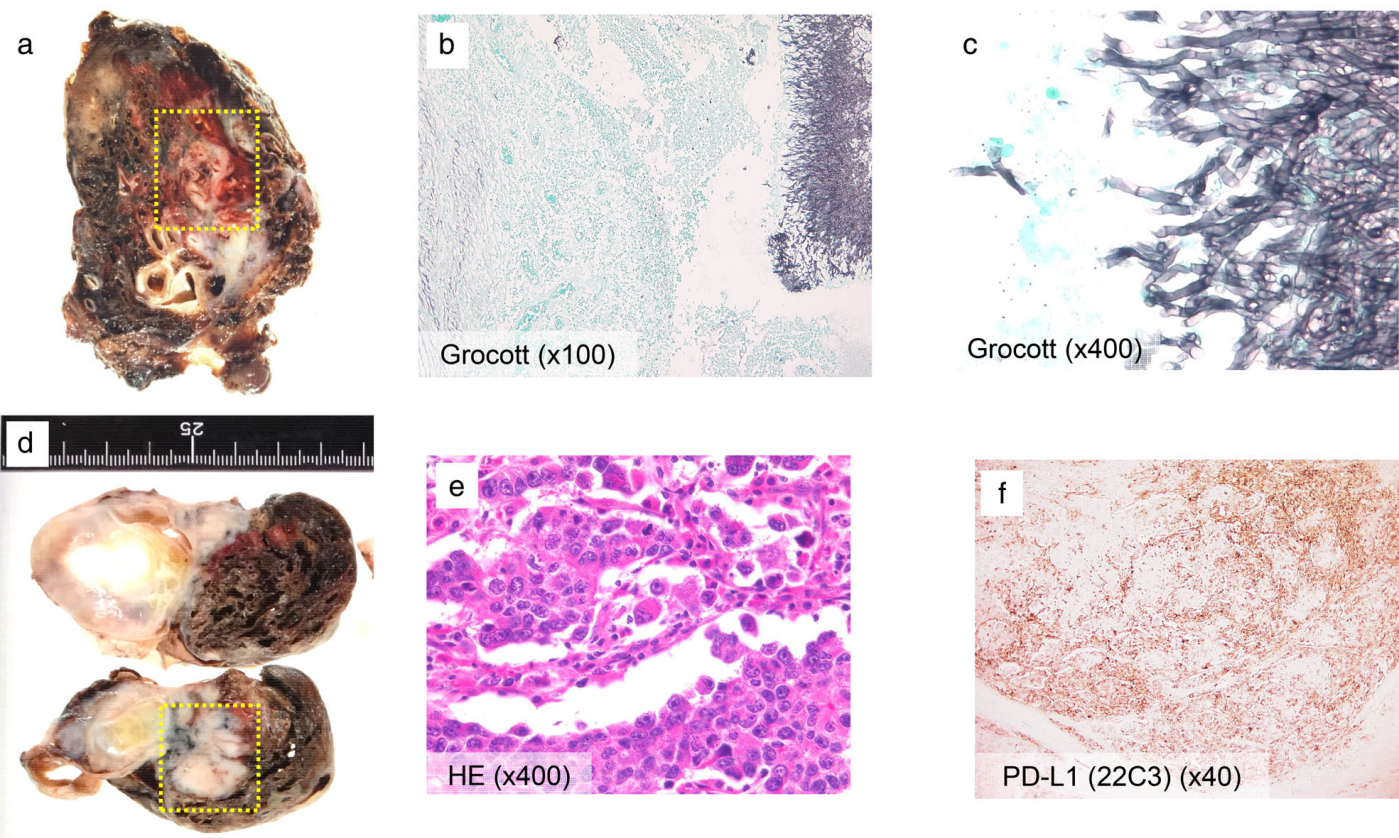
(**a**) The resected right upper lobe. (**b**, **c**) Grocott's stain showed Y shape filamentous fungi in the cavity. (**d**) The resected left upper lobe. (**e**) Hematoxylin and eosin (H&E) stain revealed conspicuous nucleoli. (**f**) PD‐L1 stain (22C3 antibody) showed the tumor proportion score (TPS) was 60% (TPS high expression).

Therefore, we conducted a pathological confirmation by surgical resection. First, as initial surgery, a wedge resection of the right upper lobe was performed to prevent pulmonary hemorrhage. Additionally, we performed a wedge resection of the left upper lobe to confirm a pathological diagnosis of the left tumor suspected of lung cancer two weeks after initial surgery. From the right lung specimen, there were necrotic changes and massive inflammatory cell invasion around cavity wall. Grocott stain showed typical Y shape filamentous fungi were present in the cavity, identified as *Aspergillus fumigatus* (Fig 2a–c). From the left lung specimen, lung adenocarcinoma was diagnosed pathologically. PD‐L1 stain (22C3 antibody) showed tumor proportion score (TPS) as 60% immunohistochemically (Fig 2d–f). No oncogenic driver aberrations were detected such as EGFR, ALK, ROS1 and BRAF. The patient was finally diagnosed with lung adenocarcinoma cT3N2M1c stage IVB, complicated by pulmonary aspergilloma. After surgery, we started first‐line chemotherapy (cisplatin, pemetrexed and bevacizumab). The response was stable disease (SD). However, after the fourth cycle of first‐line chemotherapy, the mediastinal lymph nodes were noted to be enlarged and the tumor markers had rapidly increased. ECOG PS had worsened from PS 1 to PS 3. We considered best supportive care, but the patient requested anti‐PD‐1 therapy. After informed consent, we commenced nivolumab as second‐line therapy at a dose of 3 mg/kg bodyweight every two weeks.

**Figure 3:**
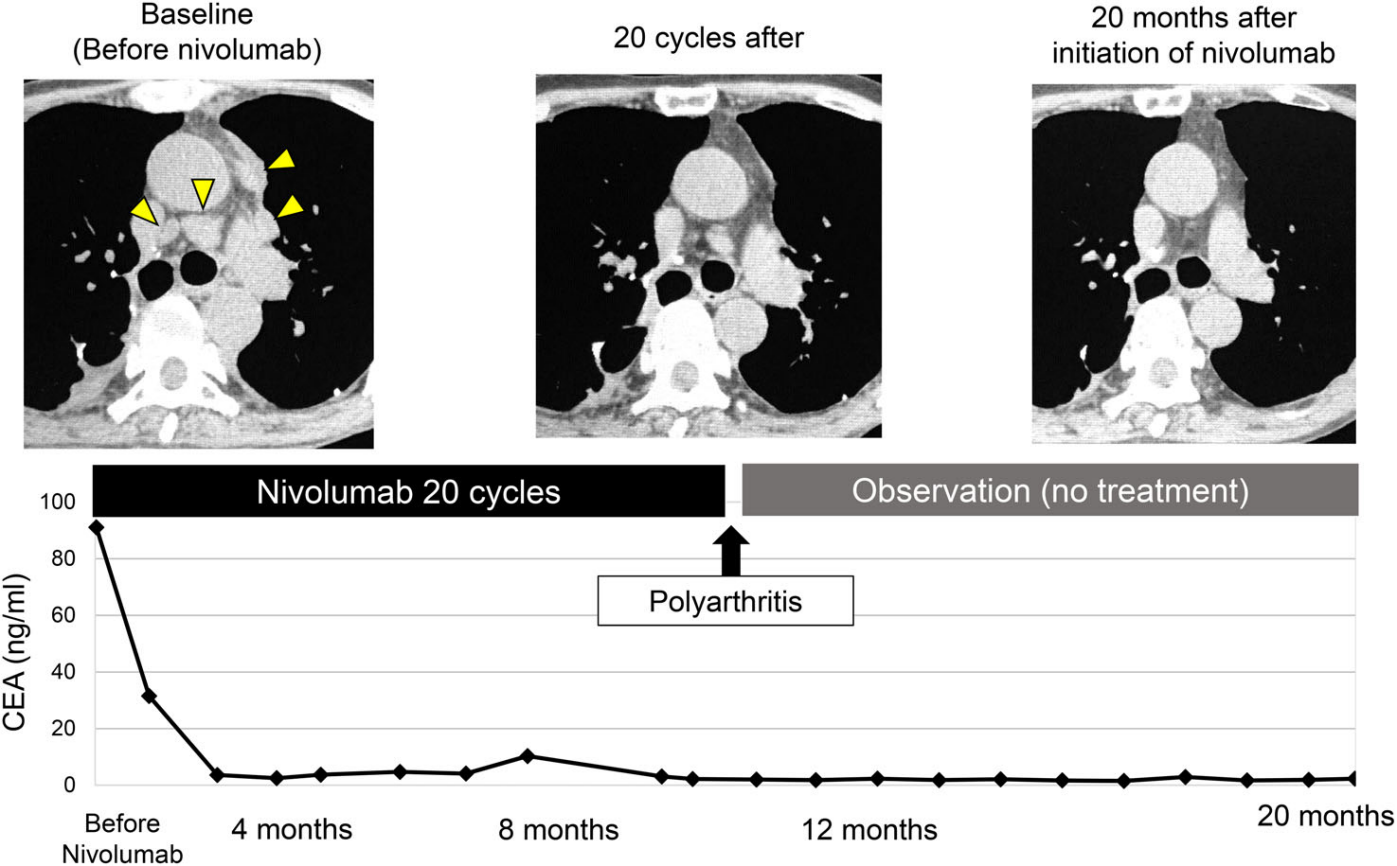
Clinical course and tumor marker (CEA) levels. After initiation of nivolumab, CEA was normal and the mediastinal lymph nodes had completely disappeared (arrows).

**Figure 4:**
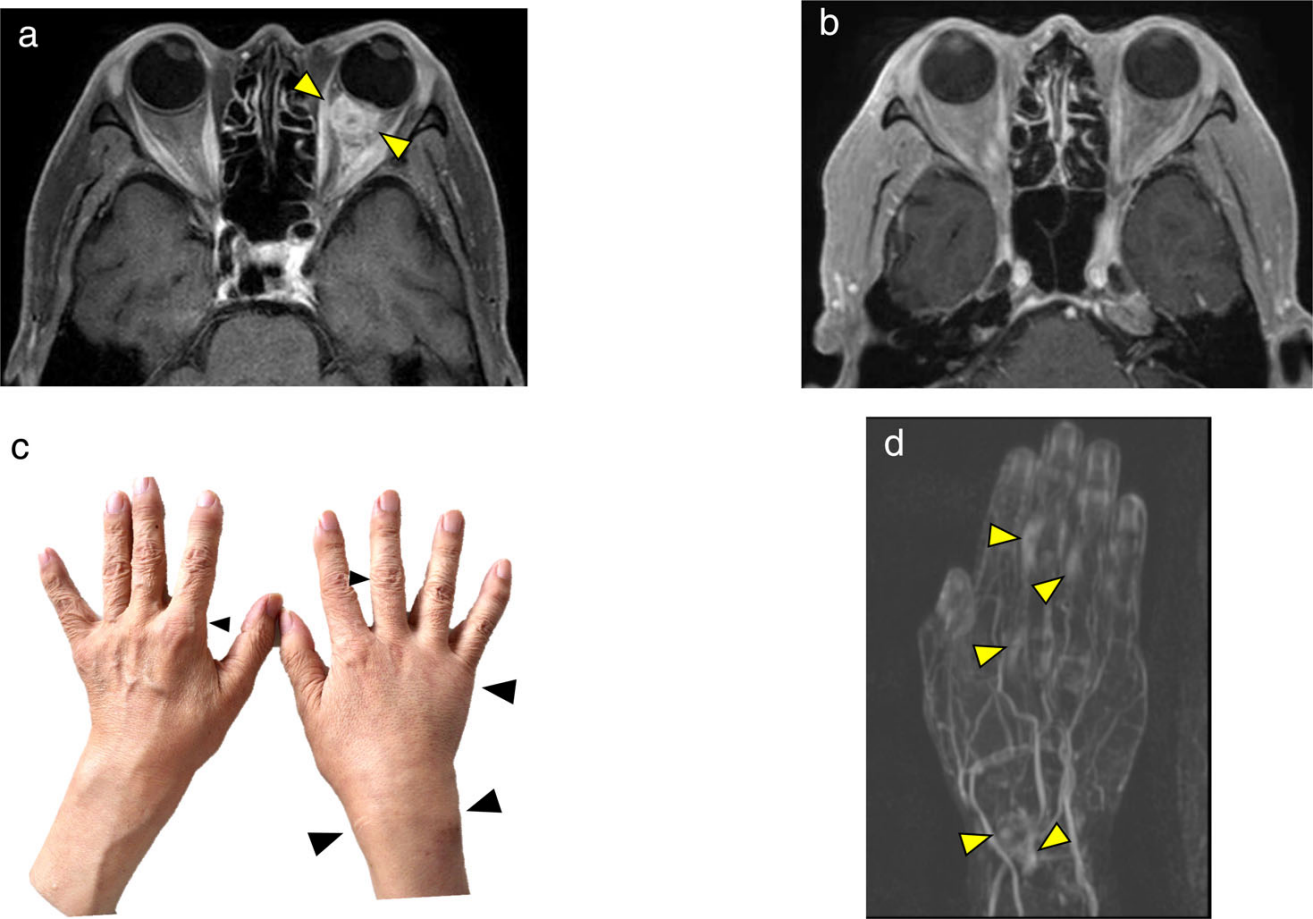
(**a**, **b**) The metastatic site in the left choroid had completely disappeared (arrows). (**c**) After 20 cycles of nivolumab, multiple joint pains and swelling occurred (left wrist and fingers, arrows). (**d**) Magnetic resonance imaging (MRI) revealed high signal intensity signs in the left wrist and fingers on dynamic gadolinium‐enhanced MRI (arrows).

After initiation of nivolumab, the mediastinal lymph nodes shrunk rapidly and tumor markers were almost normal. ECOG PS also improved from PS 3 to PS 0. The response to nivolumab was complete response (CR) (Figs 3, 4). However, after 20 cycles of treatment, polyarthritis occurred (Fig 4c), and subsequent MRI scan of the patient's hand revealed high signal intensity signs in the left wrist and fingers on gadolinium‐enhanced MRI (Fig 4d). These findings were specific for synovitis. We considered that this adverse event was an immune‐related adverse event (irAE). Therefore, we treated the patient with oral prednisolone (PSL) 5 mg/day and salazosulfapyridine (SASP) 1000 mg/day. After initiation of PSL and SASP, the patient's multiple joint pains and swelling improved, and subsequently he did not receive any further anticancer treatment, and a complete treatment response continued for over 20 months with good PS.

## Discussion

Here, we describe the first successful report of a patient safely treated with nivolumab for advanced stage NSCLC complicated by pulmonary aspergilloma. Nivolumab and durvalumab have previously been reported to induce acute progression of aspergillosis in a patient with NSCLC.^5^, ^6^ In contrast, we were able to safely treat the case reported here by surgical resection of pulmonary aspergilloma before nivolumab administration.

ICIs enhance host cytotoxic T cell immune systems, resulting in an antitumor effect.^1^ However, we are often concerned that ICIs might lead to serious immune inflammatory reaction in patients whose conditions are complicated by chronic infectious disease.

Pulmonary aspergillosis is classified into three categories: allergic bronchopulmonary aspergillosis (ABPA), chronic pulmonary aspergillosis (CPA) and invasive pulmonary aspergillosis (IPA).^7^ In patients with locally limited CPA, surgical resection is the main treatment option. IPA complicated by lung adenocarcinoma has been previously described.^8^ In the present case, the most important point for safe management was initial surgery involving right upper wedge resection. By surgical resection of the aspergilloma, we were able to control the hemoptysis and fungal infection. Subsequently, the patient was safely treated with nivolumab without worsening of pulmonary aspergillosis.

Another concern for this case was poor PS (PS 3). There are few reports which have investigated the efficacy and safety of nivolumab in patients with poor PS (PS 3–4).^9^, ^10^ Both of these reports revealed that poor PS was a negative predictor in patients with NSCLC, treated with nivolumab. However, in the case reported here, the main reason for administration of nivolumab was high expression of PD‐L1.

Interestingly, in our study, the patient achieved a complete response following nivolumab monotherapy and has maintained a durable response, even after the discontinuation of nivolumab due to an irAE. A recent report revealed that irAE could predict durable responders.^11^ which is compatible with the case reported here.

In summary, we were able to safely treat this patient with good efficacy with nivolumab treatment for lung adenocarcinoma complicated by pulmonary aspergilloma. To safely treat patients with locally limited CPA, surgical resection should be considered before ICI therapy.

## Disclosure

Dr Furuya has received speaker fees as honoraria from Eli Lilly Japan, Chugai, AstraZeneca, Bristol Myers Squibb, Taiho, Boehringer Ingelheim Japan, Ono pharma, and Pfizer Japan. Dr Mineshita has received research grant and speaker fees as honoraria from Chugai, AstraZeneca, Novartis pharma, Taiho, Boehringer Ingelheim Japan, Daiichi Sankyo, Astellas pharma and Pfizer Japan. No funding was received for this manuscript. The remaining authors state that they have no conflict of interest.

## References

[1] Pardoll DM . The blockade of immune checkpoints in cancer immunotherapy. Nat Rev Cancer 2012; 12 (4): 252–64. 22437870 10.1038/nrc3239 PMC4856023

[2] Borghaei H , Paz‐Ares L , Horn L *et al* Nivolumab versus docetaxel in advanced nonsquamous non‐small‐cell lung cancer. N Engl J Med 2015; 373 (17): 1627–39. 26412456 10.1056/NEJMoa1507643 PMC5705936

[3] Fujita K , Terashima T , Mio T . Anti‐PD1 antibody treatment and the development of acute pulmonary tuberculosis. J Thorac Oncol 2016; 11 (12): 2238–40. 27423391 10.1016/j.jtho.2016.07.006

[4] Oltolini C , Ripa M , Andolina A *et al* Invasive pulmonary aspergillosis complicated by Carbapenem‐resistant *Pseudomonas aeruginosa* infection during Pembrolizumab immunotherapy for metastatic lung adenocarcinoma: Case report and review of the literature. Mycopathologia 2019; 184 (1): 181–4. 30101407 10.1007/s11046-018-0291-4

[5] Uchida N , Fujita K , Nakatani K *et al* Acute progression of aspergillosis in a patient with lung cancer receiving nivolumab. Respirol Case Rep 2017; 6 (2): e00289. 29321933 10.1002/rcr2.289 PMC5756713

[6] Gupta A , Tun A , Ticona K , Baqui A , Guevara E . Invasive aspergillosis in a patient with stage III (or 3a or 3b) non‐small‐cell lung cancer treated with Durvalumab. Case Rep Oncol Med 2019; 2019: 1–4. 10.1155/2019/2178925 PMC6732593 31534809

[7] Kanj A , Abdallah N , Soubani AO . The spectrum of pulmonary aspergillosis. Respir Med 2018; 141: 121–31. 30053957 10.1016/j.rmed.2018.06.029

[8] Boyd M , Ojha S , Goyos J , Cragun WH , Rubio E . Invasive pulmonary aspergillosis and lung adenocarcinoma: Case report. Thorac Cancer 2013; 4 (2): 212–4. 28920192 10.1111/j.1759-7714.2012.00145.x

[9] Fujimoto D , Yoshioka H , Kataoka Y *et al* Efficacy and safety of nivolumab in previously treated patients with non‐small cell lung cancer: A multicenter retrospective cohort study. Lung Cancer 2018; 119: 14–20. 29656747 10.1016/j.lungcan.2018.02.017

[10] Katsura H , Suga Y , Araya T *et al* Efficacy and safety of Nivolumab in patients with advanced non‐small‐cell lung cancer and poor performance status. J Cancer 2019; 10 (10): 2139–44. 31258716 10.7150/jca.31217 PMC6584408

[11] Akamatsu H , Murakami E , Oyanagi J *et al* Immune‐related adverse events by immune checkpoint inhibitors significantly predict durable efficacy even in responders with advanced non‐small cell lung cancer. Oncologist 2020; 25 (4): e679–83. 32297443 10.1634/theoncologist.2019-0299 PMC7160399

